# Behavior of Supramolecular Polymerization of Alkynylplatinum(II) Complex in Different Compositions of DMSO and Water

**DOI:** 10.1002/asia.70184

**Published:** 2025-08-01

**Authors:** Minjoo Kim, Hyunmin Han, Sung Ho Jung, Jong Hwa Jung

**Affiliations:** ^1^ Department of Chemistry Gyeongsang National University (GNU) 501 Jinju‐Daero Jinju 52828 S. Korea; ^2^ Research Institute of Advanced Chemistry Gyeongsang National University (GNU) 501 Jinju‐Daero Jinju 52828 S. Korea

**Keywords:** Pt–Pt interaction, Solvent‐dependent assembly, Supramolecular polymer

## Abstract

We report the self‐assembling behavior of a terpyridine‐based alkyne platinum(II) complex (*R*‐**L**‐Pt‐BP) under varying composition ratios of DMSO and H_2_O. The resulting self‐assemblies exhibited distinct photoluminescence and circular dichroism (CD) properties depending on the solvent conditions. Notably, strong positive CD signals were observed for the self‐assembled *R*‐**L**‐Pt‐BP in DMSO and H_2_O (2:8 v/v), accompanied by a red‐shift in emission wavelength. In contrast, a negative CD signal was observed for the self‐assembled *R*‐**L**‐Pt‐BP in DMSO and H_2_O (5:5 v/v), along with a blue shift in photoluminescence (PL). The self‐assembly formed in DMSO and H_2_O (2:8 v/v) followed a cooperative model characterized by a nucleation–elongation mechanism, whereas those formed in DMSO and H_2_O (5:5 and 8:2 v/v) adhered to an isodesmic model. Thermodynamic parameters were determined using EQ model of the heating curves. The Gibbs free energy (Δ*G*) of the self‐assemblies formed in DMSO and H_2_O (5:5 and 2:8 v/v) was found to be higher than that formed in DMSO and H_2_O (8:2 v/v).

## Graphical abstract

The self‐assembly of *R*‐**L**‐Pt‐BP in DMSO/H_2_O showed solvent‐dependent CD and photoluminescence (PL). A cooperative mechanism with red‐shifted emission and higher Δ*G* appeared in 2:8 mixtures; whereas 5:5 and 8:2 followed an isodesmic model with opposite CD signals.

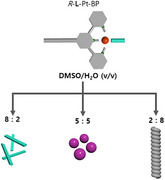

## 1 Introduction

Supramolecular self‐assembly offers an efficient approach for the miniaturization and enhancement of functional devices by leveraging various noncovalent interactions, such as van der Waals forces, coordination bonds, hydrophobic/hydrophilic interactions, electrostatic forces, hydrogen bonds, and halogen bonds.^[^ ^1^, ^2^, ^3^, ^4^, ^5^, ^6^, ^7^, ^8^, ^9^, ^10^, ^11^, ^12^, ^13^ ^]^ Due to the diversity of these noncovalent interactions, supramolecular polymeric systems can exhibit various supramolecular structures. Furthermore, the dynamic nature of noncovalent interactions imparts supramolecular polymeric systems with a variety of fascinating properties and functions, such as reversibility, adaptability, self‐healing, and stimuli‐responsiveness.^[^ ^14^, ^15^, ^16^, ^17^, ^18^, ^19^, ^20^, ^21^, ^22^, ^23^, ^24^, ^25^, ^26^ ^]^

Platinum(II) complexes, with their d^8^ electronic configuration, are widely recognized for their remarkable stability and versatile spectroscopic properties, which enable the formation of self‐assembled nanostructures such as fibers, tubes, and spheres.^[^ ^27^, ^28^, ^29^, ^30^, ^31^, ^32^, ^33^, ^34^, ^35^, ^36^, ^37^, ^38^ ^]^ In particular, square‐planar platinum(II) complexes with minimally steric ligands exhibit unique electronic characteristics and the ability to form various solid‐state structures. The fascination with platinum(II) systems largely arises from Pt···Pt interactions, which significantly influence their spectroscopic and luminescent properties by altering Pt–Pt distances.^[^ ^27^, ^28^, ^29^, ^30^ ^]^ For instance, the Yam and Chen groups have investigated alkynyl terpyridine‐based tridentate ligands in homo‐metal complex systems, emphasizing their exceptional photophysical properties and potential applications in molecular recognition, pH sensing, and biomolecular labeling.^[^ ^31^, ^32^, ^33^ ^]^ Notably, Yam and colleagues have made substantial contributions by elucidating the relationship between self‐assembly dynamics and structure–property correlations in the supramolecular polymerization of alkynylplatinum(II) terpyridine complexes.^[^ ^34^, ^35^, ^36^, ^37^, ^38^ ^]^ In particular, they demonstrated the dependence of morphology and formation mechanisms on the composition of organic solvent and water in supramolecular polymerization. Thus, investigating the influence of varying solvent compositions is crucial for a deeper understanding of the mechanisms underlying supramolecular polymerization.^[^ ^39^, ^40^ ^]^ In this study, we report the dependence of morphology, photoluminescent properties, and thermodynamic parameters of supramolecular polymers of *R*‐**L**‐Pt‐BP on varying compositions of DMSO and water. Additionally, the relationship between photoluminescent properties and chiral molecular arrangement was elucidated during the formation of supramolecular polymers.

## 2 Results and Discussion

### 2.1 Synthesis and Characterization of Ligand

**Figure 1:**
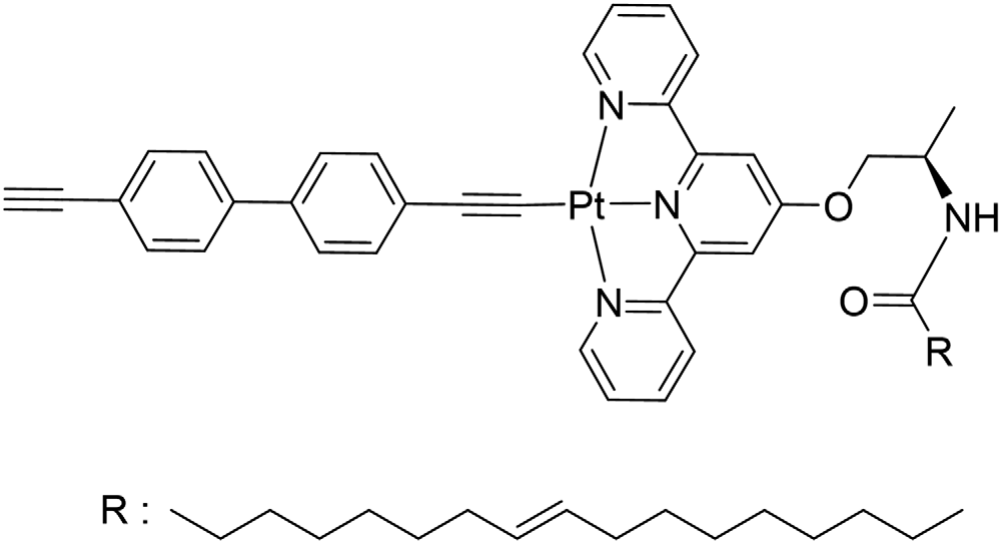
Chemical structures of terpyridine‐based *R*‐**L**‐Pt‐BP.

As illustrated in Figure 1, each chiral terpyridine ligand was synthesized individually. For instance, the chiral precursor **3*R*** was prepared using a previously reported method (Scheme S1). Compound **2*R***, which contains a trans‐double bond in the alkyl chain, was obtained by treating **3*R*** with oleoyl chloride and triethylamine in toluene. Subsequently, **L**‐Pt‐Cl was synthesized by reacting **2*R*** with dichloro(1,5‐cyclooctadiene)platinum(II) in a water and methanol mixture. Then, finally, desired product *R*‐**L**‐Pt‐BP was synthesized by treatment of **L**‐Pt‐Cl and diisopopyethylamine in DCM. All products were fully characterized by elemental analysis, NMR, IR, and HR ESI mass spectrometry (Supporting Information).

### 2.2 Photophysical Properties of *R*‐**L**‐Pt‐BP at Different Composition Ratios of DMSO and H_2_O

**Figure 2:**
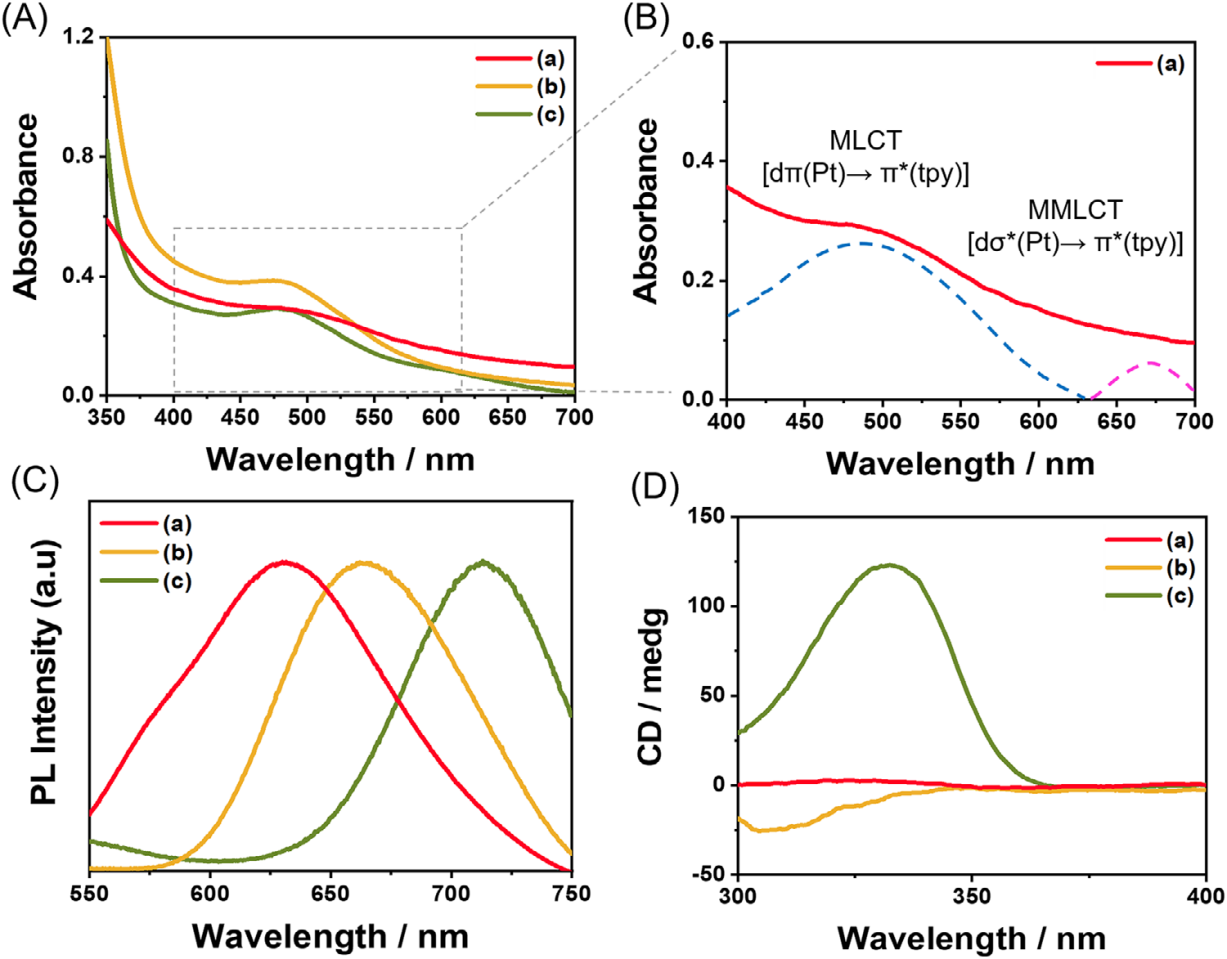
A, B) UV–vis; C) PL; and D) CD spectra of *R*‐**L**‐Pt‐BP (6.0 mM) in a different ratios of DMSO:H_2_O (v/v): a) DMSO:H_2_O (8:2 v/v), b) DMSO:H_2_O (5:5 v/v), and c) DMSO:H_2_O (2:8 v/v) after 1 day.

To study the spectroscopic properties, we first observed the UV–vis spectra of *R*‐**L**‐Pt‐BP (6.0 mM) in different composition ratios of DMSO and H_2_O (10:0 → 2:8 v/v). In pure DMSO, *R*‐**L**‐Pt‐BP exhibited similar absorption spectra with intense high‐energy absorption bands at approximately 350 nm and lower‐energy absorption bands at approximately 480 nm (Figure 2A and Figure S1). The high‐energy absorption bands were assigned to the intraligand [π→ π*] transition of terpyridine. In contrast, the lower‐energy bands were attributed to the metal‐to‐ligand charge transfer (MLCT) [dπ(Pt) → π* (terpyridine)] transition, along with a ligand‐to‐ligand charge transfer (LLCT) [π(terpyridine) → π* (terpyridine)] character. However, the absorbances at approximately 350 and 480 nm decreased in a mixed DMSO and H_2_O solution. Specifically, in DMSO and H_2_O mixture (8;2, 5:5, and 2:8 v/v) (Figure 2A,B), the absorption maxima at the high‐energy absorption band exhibited a blue shift, whereas the absorption maxima at the lower‐energy absorption band exhibited a red shift. Additionally, a weak absorption band at approximately 570 ∼ 600 nm, corresponding to metal–metal ligand charge transfer (MMLCT), was observed. These findings indicate that *R*‐**L**‐Pt‐BP forms self‐assemblies through π–π interactions and MMLCT in the mixed DMSO and H_2_O solution. The photoluminescence (PL) spectra of *R*‐**L**‐Pt‐BP were measured across varying DMSO‐to‐H_2_O composition ratios (10:0 → 2:8 v/v) (Figures 2C and S2). In pure DMSO, *R*‐**L**‐Pt‐BP exhibited a strong emission band at approximately 560 nm, attributed to the MLCT of the monomeric species. Conversely, in a DMSO and H_2_O mixture (2:8 v/v), the emission band shifted to around 650 nm as the H_2_O content increased (e.g., DMSO and H_2_O = 5:5 v/v) (Figure 2C), accompanied by enhanced emission intensity. These results suggest that MMLCT transitions, facilitated by Pt···Pt interactions in the 5:5 v/v DMSO‐H_2_O mixture, play a more significant role in self‐assembly formation compared to the 2:8 v/v mixture.

To investigate whether the supramolecular polymer of *R*‐**L**‐Pt‐BP adopts a helical or non‐helical structure, circular dichroism (CD) spectra were recorded under conditions identical to those used for UV/vis measurements (Figure 2D). The self‐assembled *R*‐**L**‐Pt‐BP (6.0 mM) formed in a mixed DMSO and H_2_O (8:2 v/v) initially exhibited a positive CD signal at approximately 320 nm (Figure S4A). However, the CD signal became silent after one day (Figure 2D), which may be attributed to a transition from a chiral to an achiral molecular arrangement. In contrast, an intense positive CD signal at 330 nm was observed in the 2:8 v/v DMSO‐H_2_O mixture (Figure 2D). This strong CD signal, originating from the π → π* transition of the terpyridine moiety, indicates the formation of a supramolecular polymer with right‐handed helicity under these conditions. Interestingly, at the initial stage, the positive CD signal of the self‐assembly formed in a mixed DMSO and H_2_O (5:5 v/v) transformed into a negative signal after one day (Figures 2D and S4B). These findings indicate that the initial positive CD signal corresponds to a kinetically favored product, whereas the negative CD signal reflects the thermodynamically stable product. Several reports in the literature have documented such CD signal inversion behavior under varying solvent conditions.^[^ ^41^, ^42^, ^43^ ^]^ The observed differences in CD response between the two solvent systems can be attributed to variations in H_2_O content. In the 2:8 (v/v) mixture, the higher proportion of polar water molecules likely enhances the solvation around the hydrophilic terpyridine–Pt(II) complex ion, thereby stabilizing the kinetically favored right‐handed helical structure. In contrast, in the 5:5 (v/v) mixture, reduced water content may lead to weaker solvation effects, facilitating a rearrangement into the thermodynamically preferred left‐handed helical structure, as reflected by the negative CD signal. Moreover, the bisphenylacetylene moiety in *R*‐**L**‐Pt‐BP may significantly influence the inversion behavior of the CD signal by altering the supramolecular packing mode and chiral expression of the assembled structures.

### 2.3 Morphology Observation

**Figure 3:**
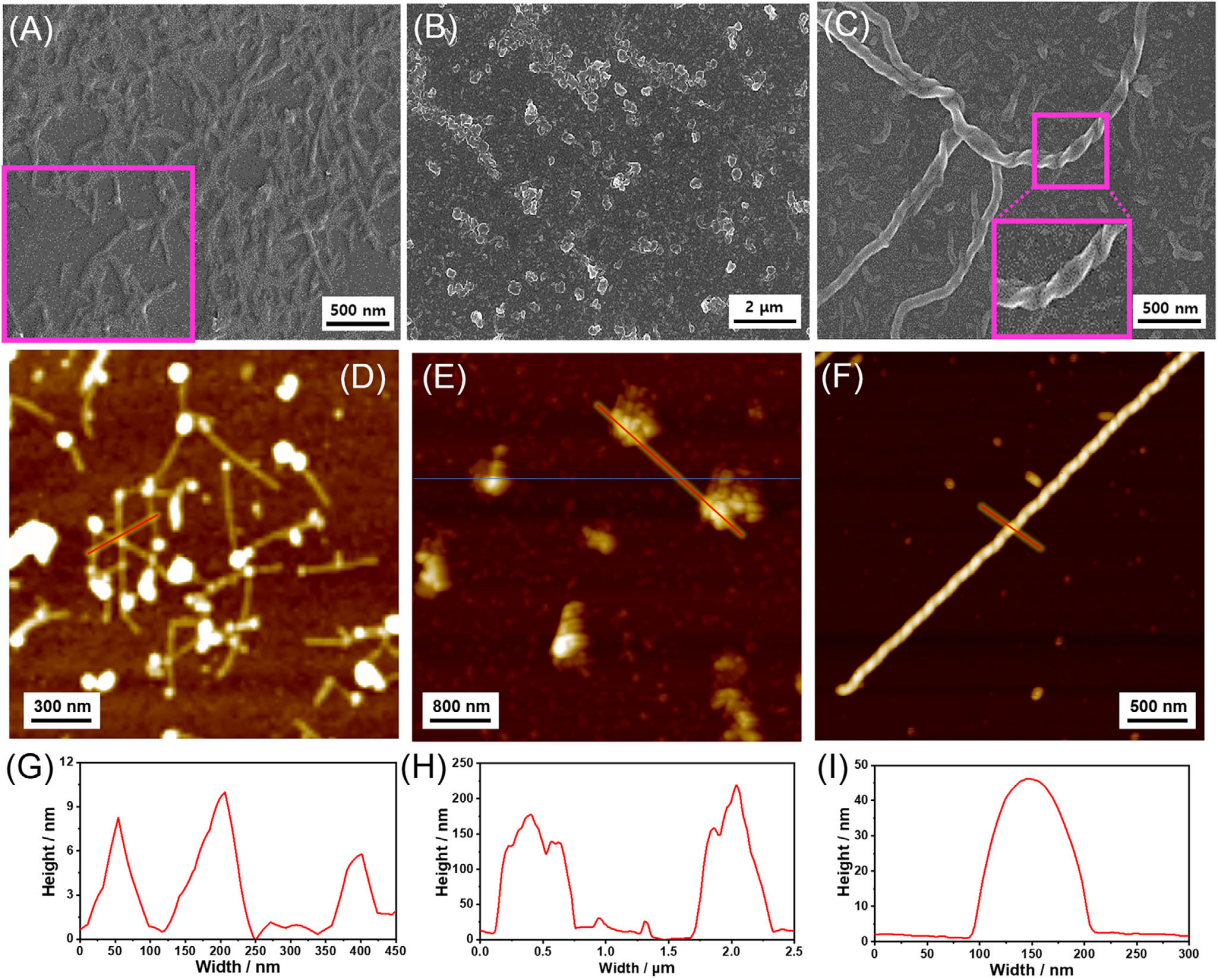
A–C) SEM, (D–F) AFM images, and G–I) height profiles of *R*‐**L**‐Pt‐BP (6.0 mM) in a different ratios of DMSO:H_2_O (v/v): (A, D, and G) DMSO:H_2_O (8:2 v/v), (B, E, and H) DMSO:H_2_O (5:5 v/v), and (C, F, and I) DMSO:H_2_O (2:8 v/v).

The morphologies of self‐assembled *R*‐**L**‐Pt‐BP (6.0 mM) formed in varying compositions of DMSO and H_2_O were analyzed using scanning electron microscopy (SEM) and atomic force microscopy (AFM). The SEM and AFM images of self‐assembled *R*‐**L**‐Pt‐BP in DMSO and H_2_O (8:2 v/v) revealed fiber‐like structures with a height of approximately 6–9 nm (Figure 3A,D,G). In contrast, the self‐assembled *R*‐**L**‐Pt‐BP in DMSO and H_2_O (2:8 v/v) exhibited helical fibers with a height of approximately 40–50 nm (Figure 3C,F,I). Interestingly, the helical fibers transitioned into spherical structures with a height of approximately 180–220 nm in DMSO and H_2_O (5:5 v/v) (Figure 3B,E,H). This morphological transformation, induced by changes in solvent composition, corresponds to the observed variations in CD and PL properties.

### 2.4 Behavior of Supramolecular Polymerization

**Figure 4:**
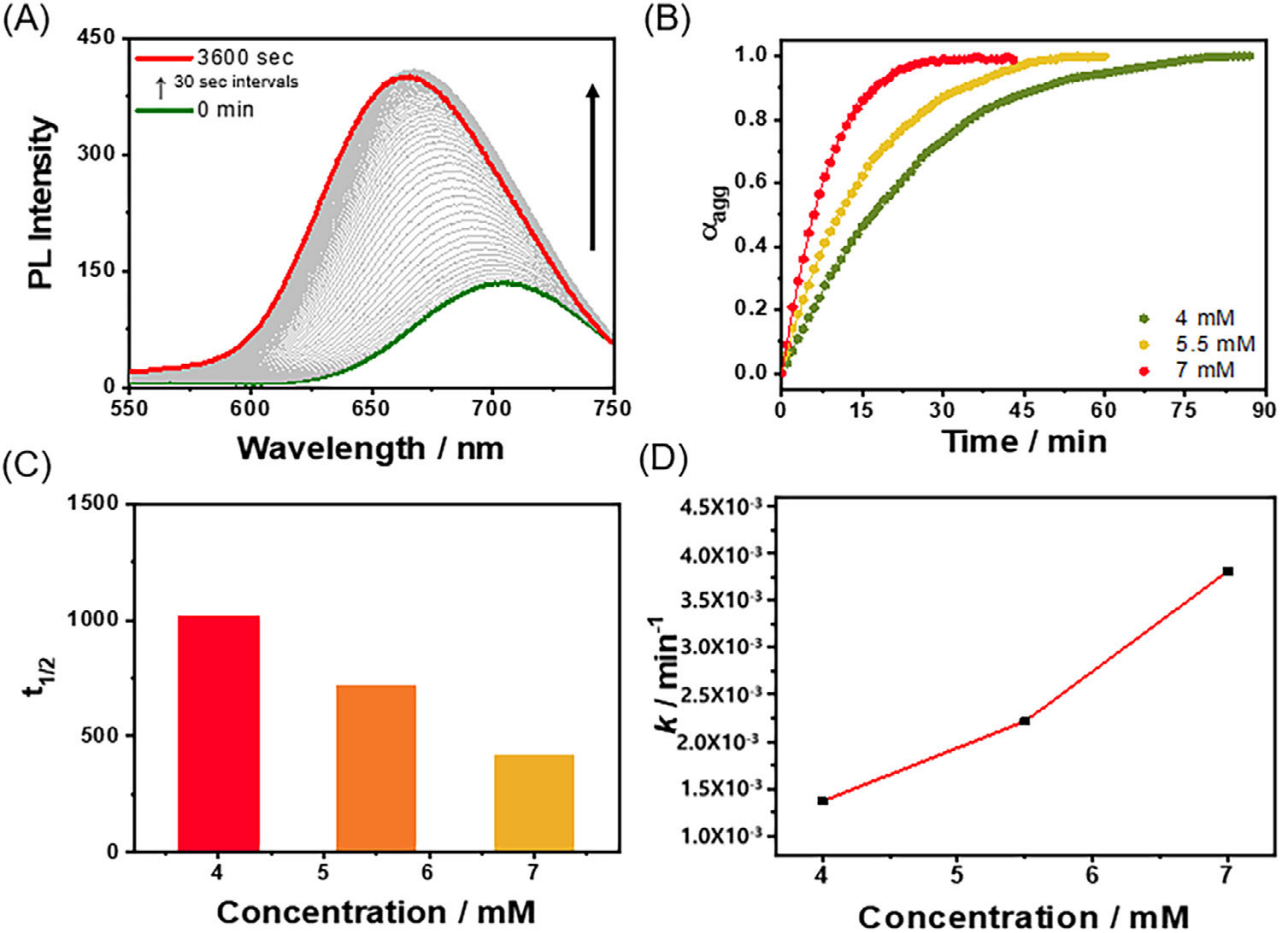
A) Time‐dependent PL spectra of *R*‐**L**‐Pt‐BP (4.0 mM, *λ*_ex_ = 420 nm) in DMSO:H_2_O (2:8 → 5:5 v/v) at 298 K. B) Plots for α_agg_ at 665 nm versus time at different concentrations of *R*‐**L**‐Pt‐BP (4.0–7.0 mM). C) Time at which 50% of final PL intensity versus concentrations of *R*‐**L**‐Pt‐BP at 298 K. D) Plot for rate constant versus different concentration of *R*‐**L**‐Pt‐BP.

To elucidate the role of solvent composition in the self‐assembly process, time‐dependent PL spectral changes of self‐assembled *R*‐**L**‐Pt‐BP (4.0 mM) in a DMSO and H_2_O mixture (2:8 v/v) at 298 K were monitored upon the incremental addition of DMSO to achieve a 5:5 v/v composition ratio. As shown in Figure 4A, the emission band initially centered at approximately 700 nm gradually shifted to 650 nm with a concurrent increase in intensity. The time‐dependent PL spectral changes exhibited a non‐sigmoidal sharp profile (Figure 4B), consistent with an “isothermal model” for self‐assembly formation. Furthermore, the rate constant (1.37 × 10^−3^–3.81 × 10^−3^) for self‐assembly increased with rising concentrations of *R*‐**L**‐Pt‐BP (4.0–7.0 mM) at 298 K, whereas the half‐life (t₁/_2_) decreased proportionally with concentration. These findings suggest that the self‐assembly of *R*‐**L**‐Pt‐BP proceeds via an “on‐pathway” mechanism, demonstrating composition‐dependent control over the assembly kinetics and morphology.

Based on the CD, PL, and SEM observations, in the DMSO and H_2_O (2:8 v/v) mixture, the self‐assembled *R*‐**L**‐Pt‐BP adopts a helical fiber structure, characterized by an ordered molecular arrangement driven by intermolecular interactions such as π–π stacking and weak Pt···Pt interactions. Upon increasing the DMSO content to a 5:5 v/v ratio, this helical fiber undergoes a transformation into a spherical structure with a relatively disordered molecular arrangement. Compared to the self‐assembly observed in the 2:8 v/v mixture, this transformation follows an on‐pathway mechanism. The resulting spherical structure exhibits stronger metal–metal‐to‐ligand charge transfer (MMLCT) properties, attributed to enhanced Pt···Pt interactions. Two key observations emerge from the compositional change of the DMSO and H_2_O mixture: at a lower DMSO composition, *R*‐**L**‐Pt‐BP forms a helical fiber structure with weak PL emission. In sharp contrast, at a higher DMSO composition, *R*‐**L**‐Pt‐BP assembles into a spherical structure with significantly enhanced PL emission.

To investigate the role of intermolecular hydrogen bonding in the formation of supramolecular nanostructures, Fourier transform infrared (FT‐IR) spectroscopy was employed. The amide I band of self‐assembled *R*‐**L**‐Pt‐BP (6.0 mM) in DMSO/H_2_O mixtures (2:8 and 5:5 v/v) appeared at approximately 1624 cm^−1^ (Figure S6), indicating the formation of intermolecular hydrogen bonds. Additionally, temperature‐dependent ^1^H NMR spectroscopy was conducted in a DMSO‐d₆/D_2_O mixture (5:1 v/v) to examine structural changes (Figure S7). At 298 K, broadening of aromatic proton peaks was observed. With increasing temperature, these peaks exhibited more pronounced low‐field shifts and improved resolution, suggesting disassembly into monomeric species. Wide‐angle X‐ray diffraction (WXRD) patterns of the self‐assemblies of *R*‐**L**‐Pt‐BP formed in DMSO/H_2_O mixtures (5:5 and 2:8 v/v) revealed three distinct peaks at *d* = 5.46, 2.73, and 1.82, corresponding to a 1:1/2:1/3 ratio (Figure S8). These results suggest the formation of a lamellar structure. The *d*‐spacing value of 5.46 nm is larger than the extended molecular length of *R‐* **L**‐Pt‐BP, which is approximately 4.39 nm according to DFT calculation (Figure S9A). This indicates that the self‐assemblies formed in DMSO/H_2_O mixtures (5:5 and 2:8 v/v) adopt bilayered structures with interdigitated lamellar arrangements (Figure S9B). Notably, a small peak at *d* = 0.34 was observed in the self‐assemblies, particularly in the sample formed in DMSO/H_2_O (5:5 v/v), which is indicative of Pt···Pt interactions. Overall, the FT‐IR and ^1^H NMR spectral data, along with WXRD analysis, strongly support the conclusion that intermolecular hydrogen bonding, in concert with π–π stacking interactions, plays a critical role in the formation of these self‐assemblies.

Conclusively, the self‐assemblies prepared in three different solvent compositions—DMSO/H_2_O = 2:8, 5:5, and 8:2 (v/v)—primarily form bilayered lamellar architectures, as illustrated in Figure S9B. The self‐assembly formed in the DMSO/H_2_O (5:5 v/v) mixture consists of approximately 20 ∼ 40 layers, exhibiting a left‐handed molecular arrangement, as deduced from DFT‐optimized structures of *R‐* **L**‐Pt‐BP and the corresponding AFM height profile (Figure 3). The self‐assembly formed in DMSO/H_2_O (2:8 v/v) consists of approximately 7 ∼ 10 layers with a right‐handed molecular arrangement (Figure 3). In contrast, the self‐assembly formed in DMSO/H_2_O (8:2 v/v) is likely to consist of a single bilayer, as suggested by the observed thickness of approximately 6–9 nm in the AFM image.

### 2.5 Thermodynamic Study

**Figure 5:**
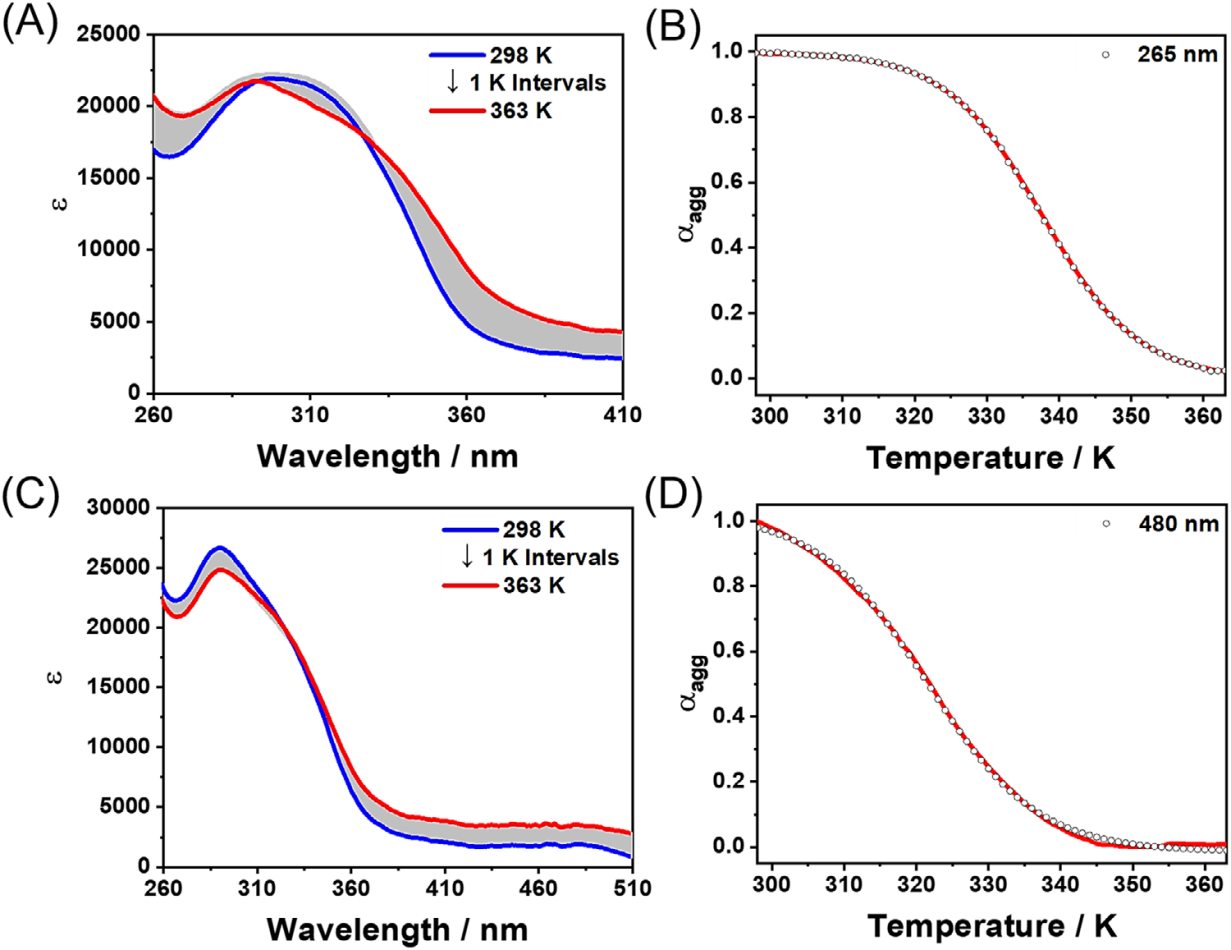
Temperature‐dependent UV–vis spectral changes of *R*‐**L**‐Pt‐BP (4.0 mM) in A) DMSO/H_2_O (2:8, v/v) and B) DMSO/H_2_O (5:5, v/v) from 298 to 363 K min^−1^. Plots for the changes of degree of *α*_agg_ versus temperatures in B) DMSO/H_2_O (2:8, v/v) and D) DMSO/H_2_O (5:5, v/v).

To determine the thermodynamic parameters of the supramolecular polymers of *R*‐**L**‐Pt‐BP, temperature‐dependent UV–vis spectra were recorded across varying compositions of DMSO and H_2_O (8:2, 5:5, and 2:8 v/v) over a temperature range of 298 K to 363 K with a ramp rate of 1 K min^−1^. The resulting heating curves were analyzed using the EQ model. For the supramolecular polymer formed in DMSO and H_2_O (2:8 v/v), the heating curve was well‐characterized by a cooperative model (Figure 5A,B). The plots of *α*_agg_ versus temperature exhibited non‐sigmoidal behavior (Figure 5B), indicating that the polymerization follows a cooperative nucleation–elongation mechanism. In contrast, the heating curves of the supramolecular polymers formed in DMSO and H_2_O (5:5 and 8:2 v/v) were better described by an isodesmic model (Figures 5C,D and S5), as evidenced by their sigmoidal shapes. Thermodynamic parameters (Δ*G*, Δ*H*, and Δ*S*) are summarized in Table S1. Notably, the Δ*G* value for the supramolecular polymer in DMSO and H_2_O (8:2 v/v) was −15.9 kJ mol^−1^ at 298 K, which was lower than those for the polymers formed in DMSO and H_2_O (5:5 and 2:8 v/v). The elongation enthalpy (Δ*H*) and elongation binding constant (*K*_e_) for this system were calculated as −114.5 kJ mol^−1^ and 1.5 × 10⁵ mol^−1^, respectively. For the supramolecular polymers formed in DMSO and H_2_O (5:5 and 8:2 v/v), the Δ*G* values were determined to be −30.9 and −29.6 kJ mol^−1^, respectively. Additionally, the cooperativity parameters (*σ* = *K*_n_/*K*_e_) in these two solvent conditions were calculated to be 1, confirming that the self‐assemblies generated in DMSO and H_2_O (5:5 and 8:2 v/v) conform to the isodesmic model.

## 3 Conclusion

In summary, we have elucidated the behavior of self‐assemblies formed in different compositions of DMSO and water. The self‐assembly prepared in DMSO/H_2_O (2:8 v/v) exhibits a strong positive CD signal but displays weak photoluminescent properties. Conversely, increasing the DMSO composition induces an inversion of the CD signal in the self‐assembly formed in DMSO/H_2_O (5:5 v/v). Moreover, the self‐assembly in DMSO/H_2_O (5:5 v/v) demonstrates enhanced PL with a blue shift. The right‐handed fibers observed in DMSO/H_2_O (2:8 v/v) transform into spherical structures upon the addition of DMSO. The self‐assembly formed in DMSO/H_2_O (2:8 v/v) follows a cooperative model, whereas the self‐assembly formed in DMSO/H_2_O (8:2 and 5:5 v/v) adheres to an isodesmic model. Both self‐assemblies comprise well‐ordered lamellar structures. This study provides valuable insights into the molecular design principles that govern self‐assembly, paving the way for advancements in the development of functional optical materials.

## Conflict of Interests

The authors declare no conflict of interest.

## Supporting information



Supporting Information

## Data Availability

The data that supports the findings of this study are available in the Supporting Information of this article.
