# Supplementary material for: Behavior of Supramolecular Polymerization of Alkynylplatinum(II) Complex in Different Compositions of DMSO and Water
Source: Chem Asian J. 2025 Aug 1;20(22):e70184. doi: 10.1002/asia.70184 (PMC12624378; doi:10.1002/asia.70184)
Supplement: Supplementary file 1 — Supporting Information [file ASIA-20-e70184-s001.docx]

**Supporting Information**

**Behavior of Supramolecular Polymerization of Alknylplatinum(II) Complex in Different Compositions of DMSO and Water**

Minjoo Kim,^[a]^ Hyeon Min Han,^[a]^ Sung Ho Jung,*^[a,b]^ and Jong Hwa Jung*^[a,b]^

^[a]^ Department of Chemistry, Gyeongsang National University, Jinju 52828, Republic of Korea

^[b]^ Research Institute of Advanced Chemistry, Gyeongsang National University, Jinju 52828, Republic of Korea

**Table of Contents**

**1. Method**

**1.1** General Characterization ·································································· S3

**1.2** SEM observation ··········································································· S3

**1.3** Circular Dichroism (UV) Studies ························································ S3

**1.4** Photoluminescence (PL) Studies ························································· S3

**1.5** Preparation of Self-Assembled Samples ················································· S4

**1.6** Thermodynamic Studies ··································································· S4

**1.7** Calculation of Rate Constant ······························································ S4

**2. Synthesis and Characterization**

**2.1** Synthesis of **3*R***··················· ···························································· S5

**2.2** Synthesis of **2*R*** ··············································································· S6

**2.3** Synthesis of **L**-Pt-Cl············· ···························································· S6

**2.4** Synthesis of *R-***L-**Pt-BP ····································································· S6

**3. Supplementary Scheme and Figures**

**3.1** Scheme S1 ···················································································· S7

**3.2** Figures S1-S9 ················································································ S8

**3.2** Table S1········ ·············································································· S14

**4. Analytical Data**

**4.1** ^1^H- and ^13^C-NMR Spectoscopy ··························································· S15

**4.2** ESI-MS Spectrometry ······································································ S19

**5. Supplementary References** ··································································· S22

1. Materials and Methods

**1.1 General**

The ^1^H and ^13^C NMR spectra were taken on a Bruker DRX 300, and Bruker DRX 500. Mass spectroscopy samples were analyzed on a JEOL JMS-700 mass spectrometer. A UV-visible spectrophotometer (Jasco V750) was used to obtain the absorption spectra. IR spectra were observed over the range 500-4000 cm^-1^, with a Thermo scientific Nicolet iS 10 instrument. Powder X-ray patterns (PXRD) were recorded on a Rigaku model NANOPIX X-ray diffractometer with a Cu K_α_ radiation source.

**1.2 SEM Observation**

FE-SEM images were observed using a Tescan (S8000). The images of samples using an accelerating voltage 10 kV and an emission current of 8 μA. Samples were prepared by dropping solutions of supramolecular nanostructure on glasses, following by spinning, drying and coating them with a thin layer of Pt to increase the contrast.

**1.3 UV-Vis Spectroscopy Studies**

The UV-Vis spectra were recorded on a Jasco V-750 UV spectrophotometer. The UV-Vis spectra were determined over the range of 250-700 nm using a quartz cell with 0.1 mm path length. Scans were taken at rate of 400 nm/min with a sampling interval of 1 nm and response time of 1 s. After adding the prepared sample to the UV cells, it was heated to 363 K (1 K/min) to form the monomeric species in UV-Vis spectroscopy.

**1.4 Photoluminescence (PL) Studies**

The photoluminescence (PL) spectra were recorded on a JASCO FP-8650 fluorescence spectrophotometer. The PL spectra were measured over the range of 250-800 nm using a quartz cell with a 10 mm path length. Scans were taken at a rate of 400 nm/min with a sampling interval of 0.5 nm and a response time of 0.5s. To investigate the supramolecular polymerization process, temperature- and time-dependent PL spectral changes were monitored with an excitation wavelength of 420 nm.

**1.5 Preparation of Self-Assembled Samples**

The stock solution of monomeric *R*-**L**-Pt-BP was prepared in DMSO, and the monomer was injected into the required volume of H_2_O to adjust the final concentration of the required choice. The final solvent composition was varied as DMSO/H_2_O (8:2, 5:5 and 2:8 v/v) to investigate the effect of solvent ratio on supramolecular polymerization.

**1.6 Thermodynamic Studies**

The molar fraction of aggregated molecules (*α*_agg_) at a certain temperature was calculated from the absorbance at 265 nm, 311nm or 480 nm in which Abs(agg) and Abs(mono) are the absorption intensities of fully aggregated (at the lowest temperature) and purely monomeric states (at the highest temperature), respectively, and Abs(T) is the absorption intensity at a given temperature (T).^[1]^

$$\alpha_{agg}=1-\frac{Abs\left( agg \right)-Abs\left( T \right)}{Abs\left( agg \right)-Abs\left( mono \right)}$$

The plot of *α*_agg_ versus temperature provides heating curves with non-sigmoidal (cooperative mechanism) shape and sigmoidal (isodesmic mechanism), which were fitted using the models proposed by Meijer *et al*. (for cooperative mechanism).^[2]^ The standard values of enthalpy (Δ*H*°), entropy (Δ*S*°), and Gibbs free energy (Δ*G*°) were calculated using EQ model.^[3]^

An elongation binding constant (*K*_e_) for aggregation at 293 K was estimated according to equation S1, from which the enthalpy change (Δ*H*), and the entropy change (Δ*S*) were determined:

$K_{e}= e^{-(\Delta H_{e}-T\Delta S)/(RT)}$ (eq. S1)

The cooperativity factor (*σ*) and nucleation binding constant (*K*_n_) for aggregation at 293 K were determined according to equation S2:

$\sigma= \frac{K_{n}}{K_{e}} {= e}^{\Delta H_{n}/(RT)}$ (eq. S2)

**1.7 Calculation of Rate Constant**

Data were obtained by converting to αagg values with PL Intensity obtained by time-dependent PL spectra measurements at aggregate formation temperatures. The rate constants *k*_1_ and *k*_2_ for the aggregation process following an isodesmic mechanism were obtained by fitting the previously described data to the F-W model.


$${[B]}_{t}={[A]}_{0}-\frac{\frac{k_{1}}{k_{2}}+{[A]}_{0}}{1+\frac{k_{1}}{k_{2}{[A]}_{0}}exp(k_{1}+k_{2}\left[ A \right]_{0})t}$$

Here [A]_0_ is initial concentration of monomeric state, [B]_t_ is concentration of polymeric state at time *t* and *k*_1_ and *k*_2_ correspond to average rate constants for nucleation and elongation steps.

2. Synthesis and Characterization

Unless otherwise noted, chemical reagents and solvents were purchased from commercial suppliers (Tokyo Chemical Industry (TCI), Sigma Aldrich) and used without further purification.

**2.1 Synthesis of *3R***

(*R*)-(−)-2-amino-1-propanol (0.126 g, 1.68 mmol) was added to a stirred suspension of KOH (0.31 g, 5.6 mmol) in anhydrous DMSO (4 mL) at 60 °C. After 60 min, 4’-chloro-2,2’:6’,2”-terpyridine (0.3 g, 1.12 mmol) in anhydrous DMSO (10 mL) was added to the mixture, which was maintained with stirring for 4 h at 70 °C. Distilled water (600 mL) was then added to the reaction mixture, and the product was extracted with CH_2_Cl_2_ (3 × 200 mL). Residual water in the CH_2_Cl_2_ was removed using Na_2_SO_4_, and the CH_2_Cl_2_ was removed using a rotary evaporator. Yield: 69.5%; IR (KBr pellet): 2366, 1579, 1561, 1468, 1440, 1405, 1353, 1204,1035, 799 cm^-1^; ^1^H NMR (300 MHz, CDCl_3_) δ 8.70 (ddd, *J* = 4.8, 1.8, 0.9 Hz, 2H), 8.62 (dt, *J* = 8.0, 1.1 Hz, 2H), 8.02 (s, 2H), 7.86 (ddd, *J* = 8.0, 7.5, 1.8 Hz, 2H), 7.34 (ddd, *J* = 7.5, 4.8, 1.2 Hz, 2H), 4.18 (m, 1H), 3.97 (dd, *J* = 9.0, 7.6 Hz, 1H), 3.46 (dddd, *J* = 10.7, 7.5, 6.5, 4.0 Hz, 1H), 1.99 (s, 2H), 1.25 (d, *J* = 6.5 Hz, 3H); ^13^C NMR (125 MHz, CDCl_3_) δ ppm 167.12, 157.16, 156.09, 149.06, 136.83, 123.87, 121.37, 107.36, 74.69, 46.27, 19.78; HR-Mass (m/z): Calculated for C_18_H_18_N_4_O [M]^+^ 306.14, Found [M+H]^+^ 307.240.

**2.2 Synthesis of *2R***

Compounds ***3R*** (0.5 g, 2 mmol), elaidic acid (0.5 g, 2 mmol), SOCl_2_ (1 mL, 20 mmol), and triethylamine (TEA, 2.5mL, 20 mmol) were added to a 50-mL flask. Anhydrous toluene (10 mL) was then injected, and the reaction mixture was stirred for 2 hours at 80 °C. Then solvents were removed using a rotary evaporator. After the crude product was partitioned in CH_2_Cl_2_/H_2_O, the organic layer was separated and dried over Na_2_SO_4_. Then, the organic solvent was removed using a rotary evaporator. The desired product was recrystallized from ethyl ether and MeOH. Yield: 52%; ^1^H NMR (300 MHz, DMSO-*d*_6_) δ 8.71 (ddd, *J* = 4.8, 1.8, 0.9 Hz, 2H), 8.62 (dt, *J* = 8.0, 1.1 Hz, 2H), 8.06 – 7.96 (m, 4H), 7.89 (d, *J* = 7.4 Hz, 1H), 7.50 (ddd, *J* = 7.5, 4.8, 1.2 Hz, 2H), 5.36 – 5.18 (m, 2H), 4.32 – 4.06 (m, 3H), 2.07 (t, *J* = 7.2 Hz, 2H), 1.85 (dd, *J* = 13.1, 5.5 Hz, 4H), 1.47 (s, 2H), 1.21 (d, *J* = 5.5 Hz, 24H), 0.84 (s, 3H); HR-Mass (m/z): Calculated for C_35_H_50_ClN_4_O_2_Pt [M]^+^ 571.401, Found [M]^+^ 571.4018 and [M+H]^+^ 572.4050.

**2.3 Synthesis of L-Pt-Cl**

Dichloro(1,5-cyclooctadiene)platinum(II) (0.18 g, 0.5 mmol) was added to a suspension of *2S-trans-***1** (0.3 g, 0.5 mmol) in mixture of 10 mL anhydrous methanol. The reaction mixture was stirred for 2 hours at 90 °C. Then solvents were removed using a rotary evaporator. Then desired product was recrystallized from methanol and ethyl ether. Yield: 71.7%; IR (KBr pellet): 3065, 2924, 2853, 1730, 1661, 1653, 1647, 1609, 1547, 1460, 1416, 1364 cm^-1^; ^1^H NMR (300 MHz, DMSO-*d*_6_) δ 8.78 (d, *J* = 5.6 Hz, 2H), 8.69 (d, *J* = 8.0 Hz, 2H), 8.49 (td, *J* = 7.9, 1.5 Hz, 2H), 8.31 (s, 2H), 8.14 (s, 1H), 7.91 (t, *J* = 6.8 Hz, 2H), 5.31 (s, 2H), 4.28 (q, *J* = 8.8, 7.5 Hz, 3H), 2.11 (td, *J* = 7.3, 2.6 Hz, 2H), 1.89 (s, 4H), 1.61 – 1.39 (m, 3H), 1.25 (d, *J* = 14.9 Hz, 27H), 0.85 (t, *J* = 6.4 Hz, 4H); HR-Mass (m/z): Calculated for C_35_H_50_ClN_4_O_2_Pt [M]^+^ 800.334, Found [M]^+^ 800.3342 and [M+H]^+^ 801.3344.

**2.3 Synthesis of *R*-L-Pt-BP**

4,4’-Diethynylbiphenyl (0.607g, 0.003mol) and anhydrous methyl chloride were added to a 50-mL flask. *S-trans-1* (0.5g, 0.0006mol), Diisopopylethylamine (0.5mL) and Copper iodide (0.01g) were then added. The reaction mixture was stirred for 6 hours with the light blocked. Then solvents were removed using a rotary evaporator. After the crude product was partitioned in CH_2_Cl_2_/H_2_O, the organic layer was separated and dried over Na_2_SO_4_. Then desired product was recrystallized from methyl chloride and ethyl acetate. Yield: 71.7%; IR (KBr pellet): 3065, 2924, 2853, 1730, 1661, 1653, 1647, 1609, 1547, 1460, 1416, 1364 cm^-1^; ^1^H NMR (300 MHz, DMSO-*d*_6_) δ 9.01 (d, *J* = 5.4 Hz, 2H), 8.67 (d, *J* = 8.0 Hz, 2H), 8.52 – 8.40 (m, 2H), 8.31 (s, 2H), 8.07 (d, *J* = 6.4 Hz, 1H), 7.86 (t, *J* = 6.8 Hz, 2H), 7.81 – 7.66 (m, 4H), 7.64 – 7.50 (m, 4H), 5.30 (t, *J* = 4.2 Hz, 2H), 4.27 (d, *J* = 13.5 Hz, 3H), 2.13 – 2.04 (m, 2H), 1.88 (s, 4H), 1.48 (s, 2H), 1.27 – 1.15 (m, 20H), 0.89 – 0.78 (m, 3H) ; HR-Mass (m/z): Calculated for C_52_H_63_N_4_O_2_Pt [M]^+^ 966.4268, Found [M]^+^ 966.2506 and [M+H]^+^ 967.2506.

3. Supplementary Schemes and Figures.


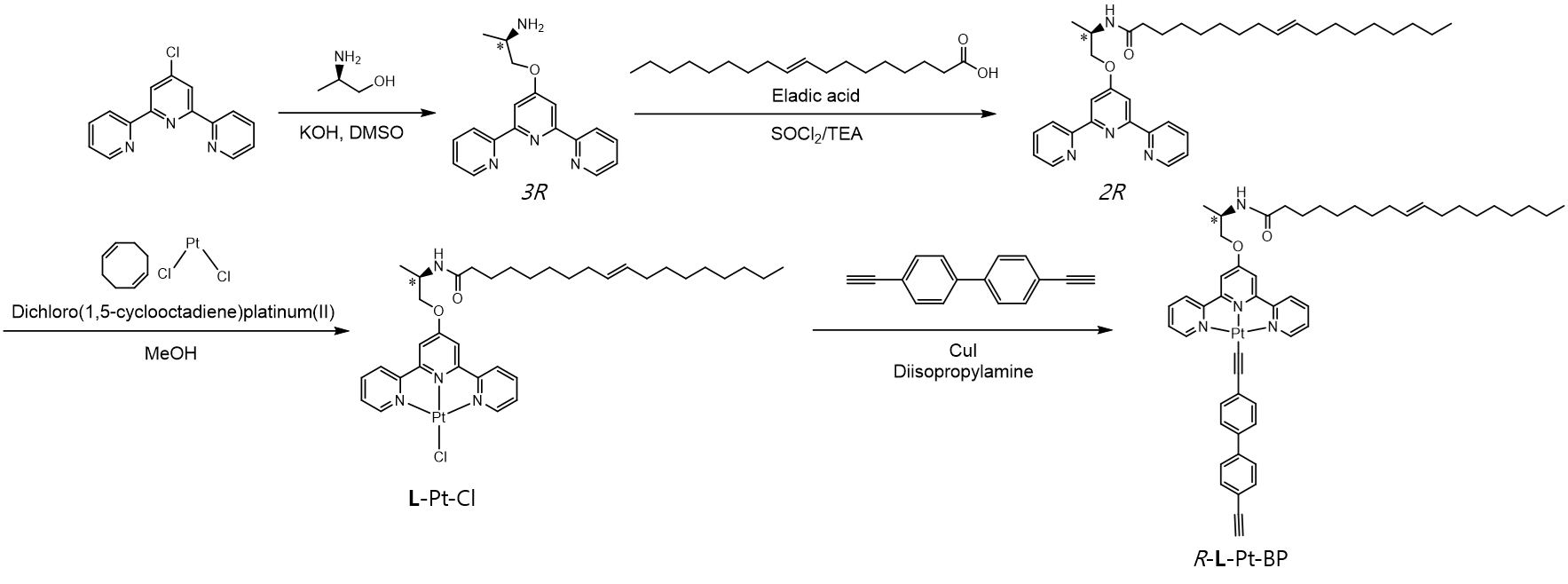


Scheme S1. Synthetic route of *R*-L-Pt-BP.


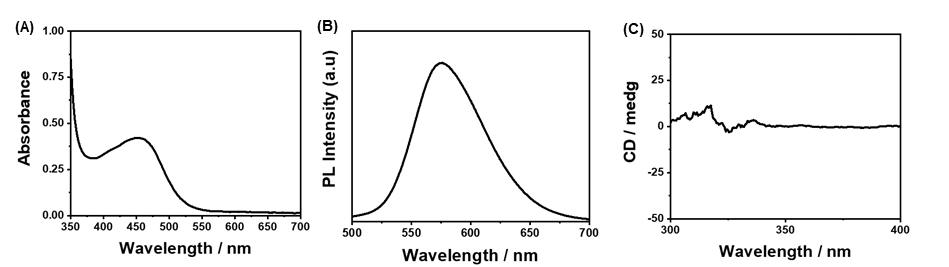


Figure S1. (A) UV-vis, (B) PL and (C) CD spectra of *R*-L-Pt-BP (6.0 mM) in DMSO only.


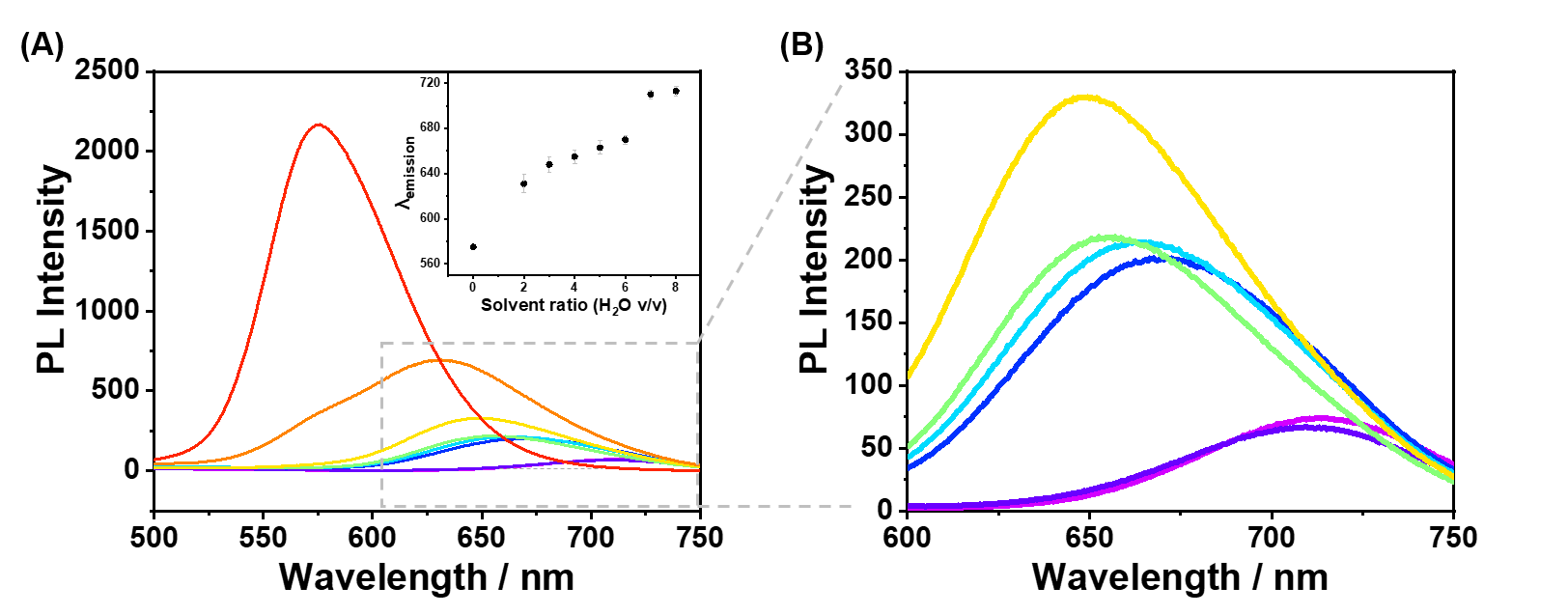


Figure S2. PL spectral changes of (A and B) *R*-L-Pt-BP (6.0 mM) in different ratios of DMSO/H_2_O (10: 0 → 2:8 v/v)
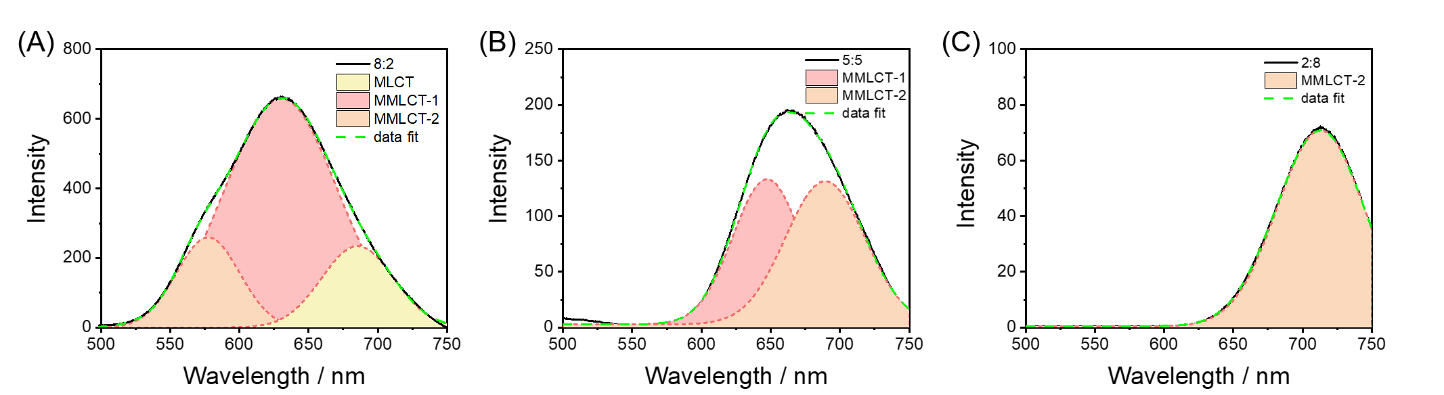


**Figure S3.** Gaussian fitting into emission bands from the spectra of *R*-**L**-Pt-BP (6.0 mM) in different composition ratios of DMSO and H_2_O (8:2 → 2:8 v/v).

**
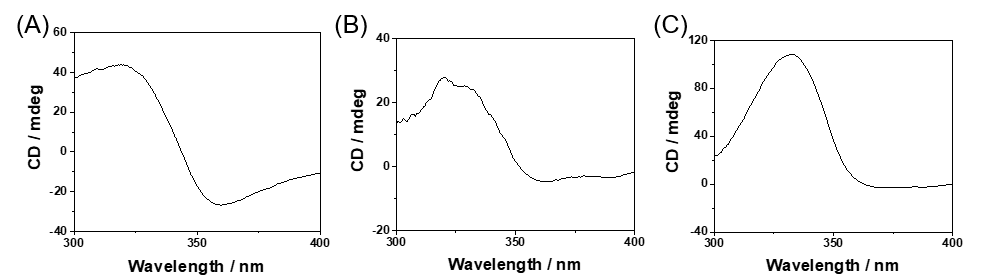
**

**Figure S4.** CD spectra of *R*-**L**-Pt-BP (6.0mM) in a mixed (A) DMSO/H_2_O (8:2 v/v), (B) DMSO/H_2_O (5:5 v/v) and (C) DMSO/H_2_O (2:8 v/v) after 5 min.

**
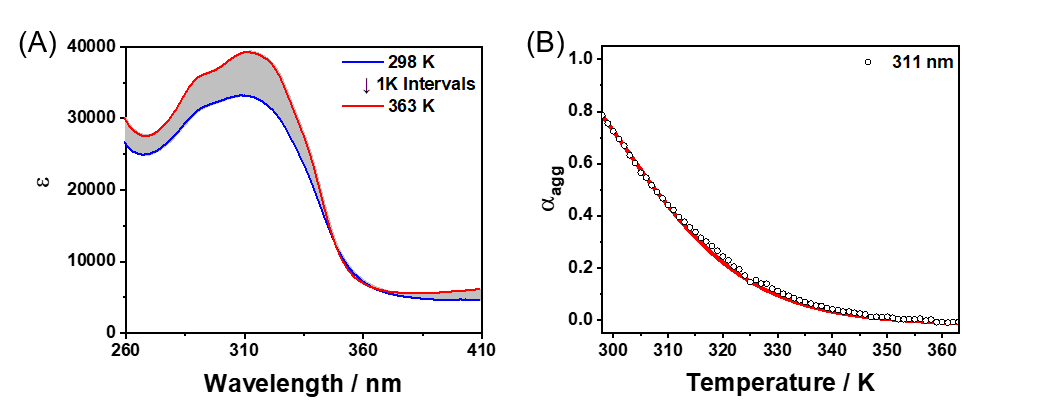
**

**Figure S5.** Temperature-dependent UV-vis spectral changes of *R*-**L**-Pt-BP (4.0 mM) in (A) DMSO/H_2_O (8:2 v/v) from 298 K to 363 K min^-1^. (B) Plots for the changes of α_agg_ of supramolecular polymer based on *R*-**L**-Pt-BP vs. temperature.

**
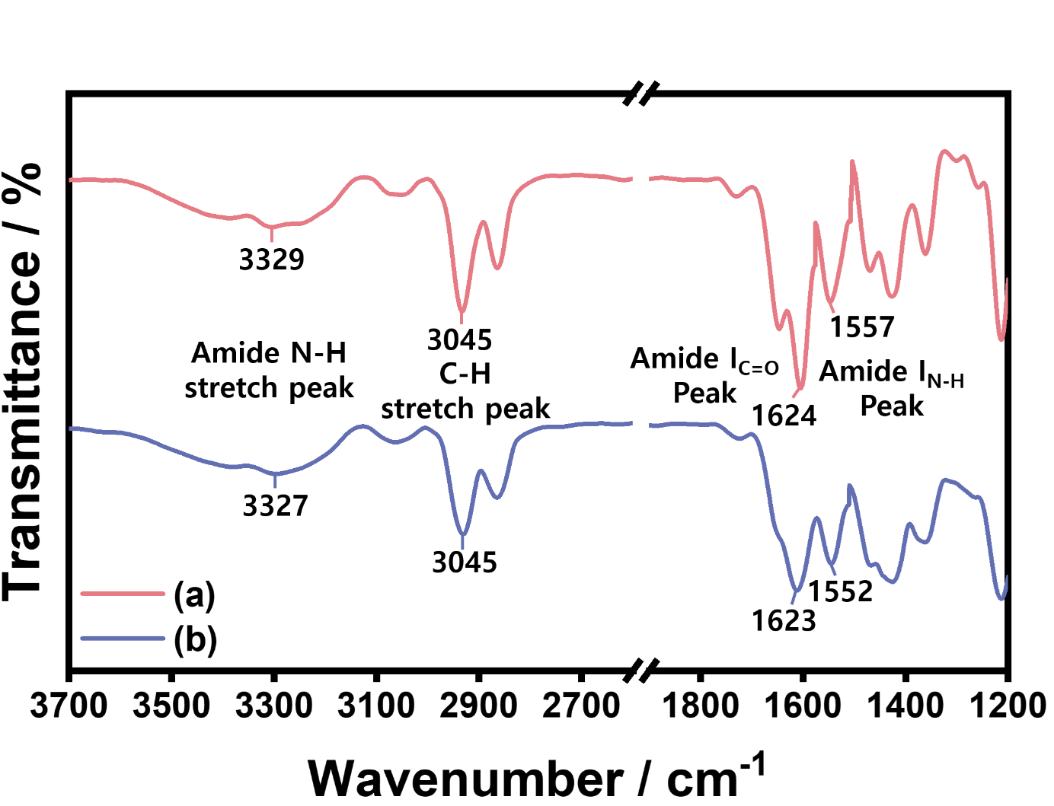
**

**Figure S6.** FT-IR spectra of *R*-**L**-Pt-BP (6.0 mM) in (a) DMSO/H_2_O (2:8 v/v) and (b) DMSO/H_2_O (5:5 v/v).**
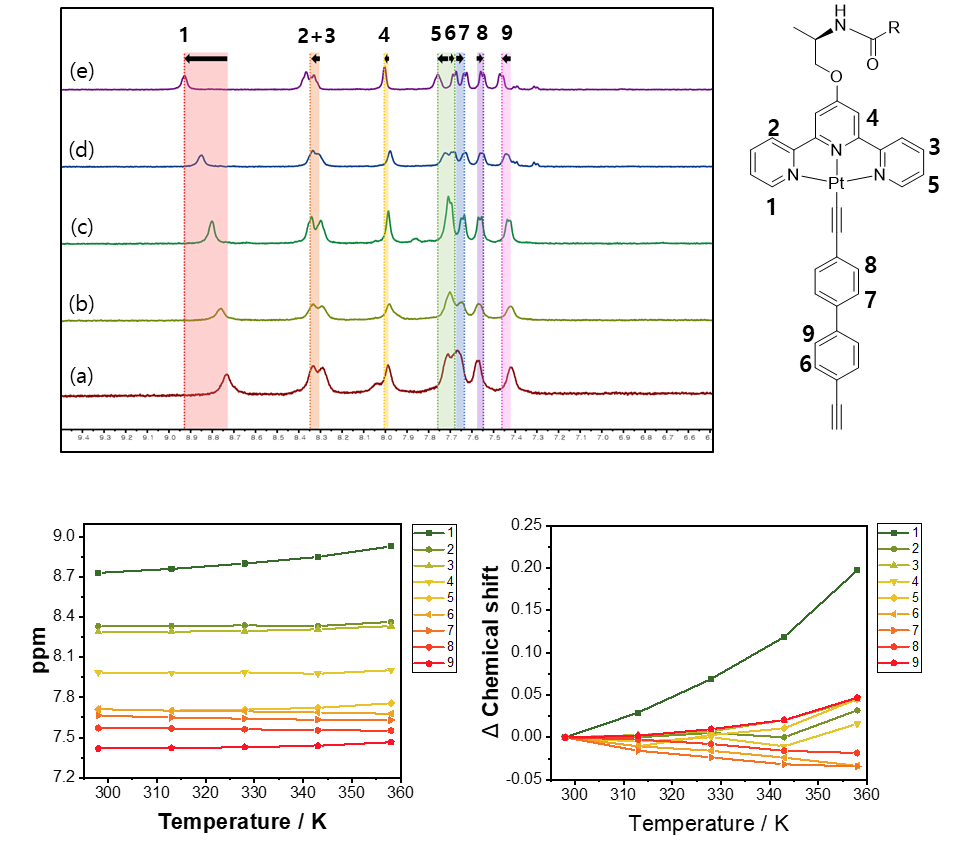
**

**Figure S7.** ^1^H-NMR spectra of *R*-**L**-Pt-BP (6.0 mM) at (a) 298 K, (b) 313 K, (c) 328 K, (d) 343 K and (e) 358 K in DMSO-*d_6_* and D_2_O (5:1 v/v).

**
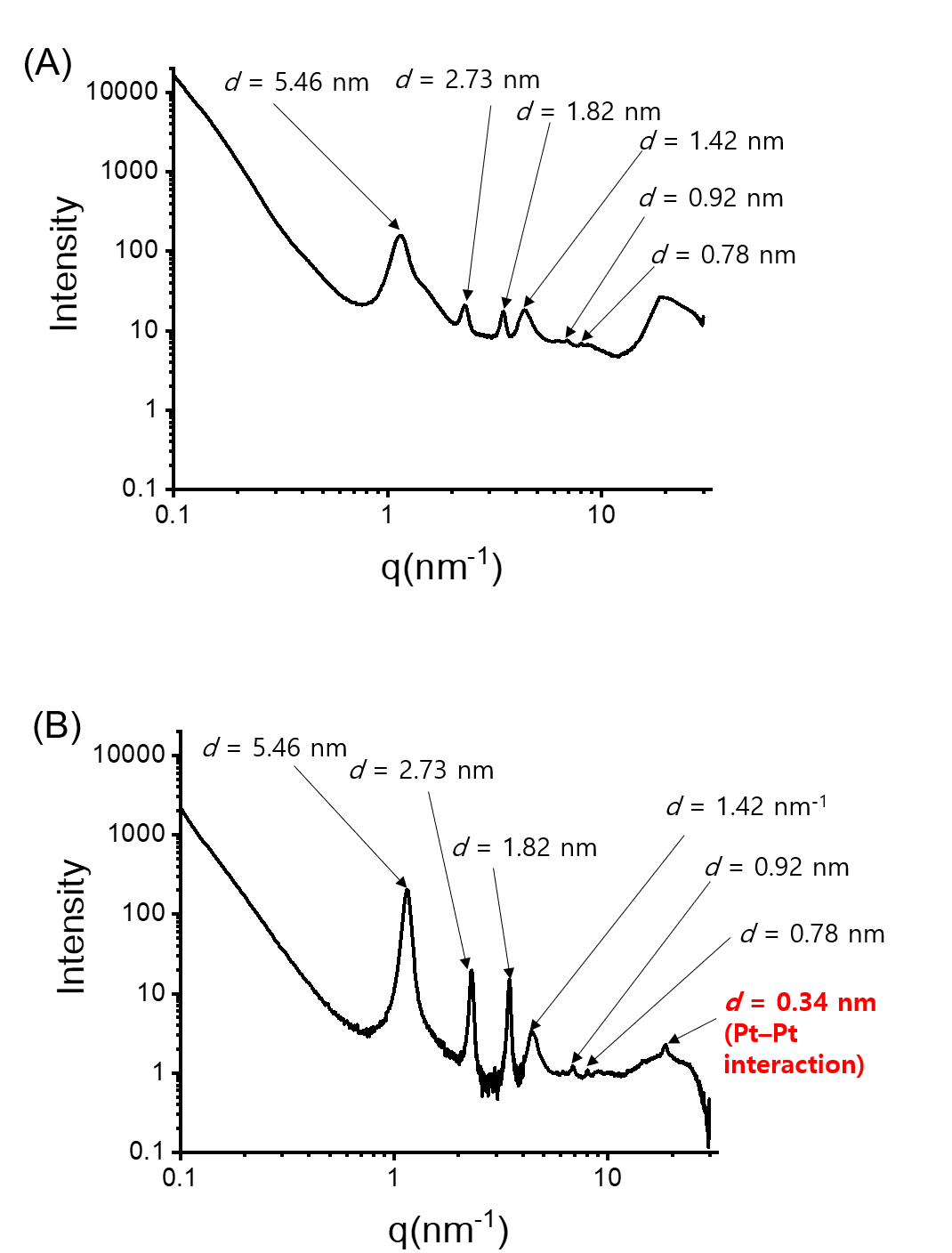
**

**Figure S8.** WAXS data of *R*-**L**-Pt-BP (6.0 mM) in (A) DMSO/H_2_O (5:5 v/v) and (B) DMSO/H_2_O (2:8 v/v).

**
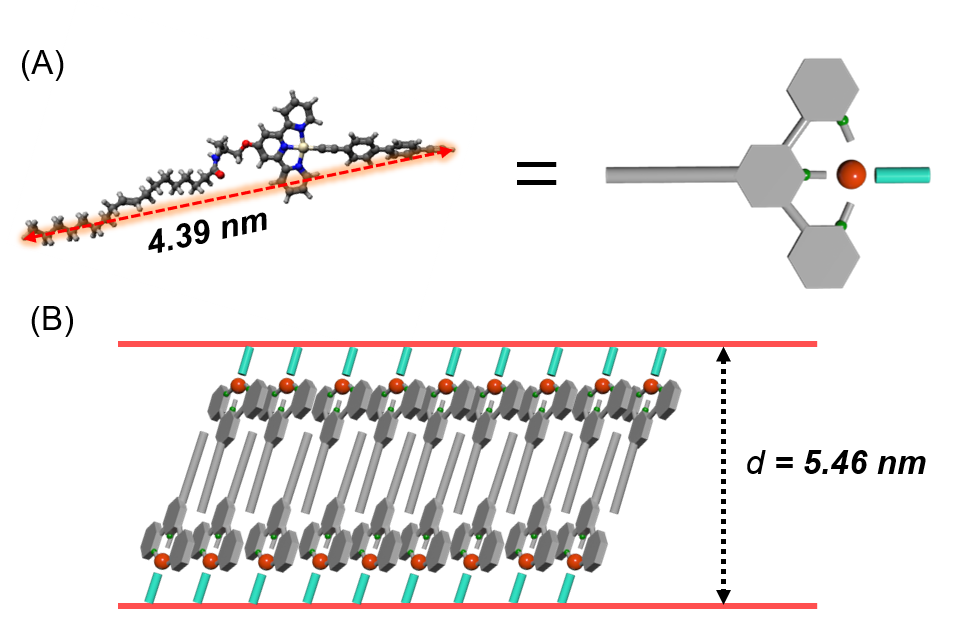
**

**Figure S9.** (A) Geometry-optimized structure of *R-***L**-Pt-BP using B3LYP/6-31G* level and (B) schematic representation of bilayer structure of the self-assembled *R*-**L**-Pt-BP.

**Table S1.** Thermodynamic parameters for the formation of *R*-**L**-Pt-BP in different composition ratios of DMSO/H_2_O (8:2 → 2:8 v/v). *K_e_* = *K* (isodesmic model)

| **DMSO/H_2_O (v/v)** | **Δ*G***  **(kJ mol^-1^)** | **Δ*H***  **(kJ mol^-1^)** | **Δ*S***  **(J K^-1^ mol^-1^)** | ***K_e_***  **(L mol^-1^)** | ***K_n_***  **(L mol^-1^)** | ***T_e_***  **(K)** | ***σ*** |
| --- | --- | --- | --- | --- | --- | --- | --- |
| 8 : 2 | -15.9 | -79.3 | -212.9 | 6.1×10 | - | - | 1 |
| 5 : 5 | -30.9 | -154.4 | -414.5 | 2.6×10^5^ | - | - | 1 |
| 2 : 8 | -29.6 | -114.5 | -285.1 | 1.5×10^5^ | 9.2×10^3^ | 340.0 | 6.1×10^-2^ |

**4. Analytic Data**

4.1 ^1^H and ^13^C NMR Spectra


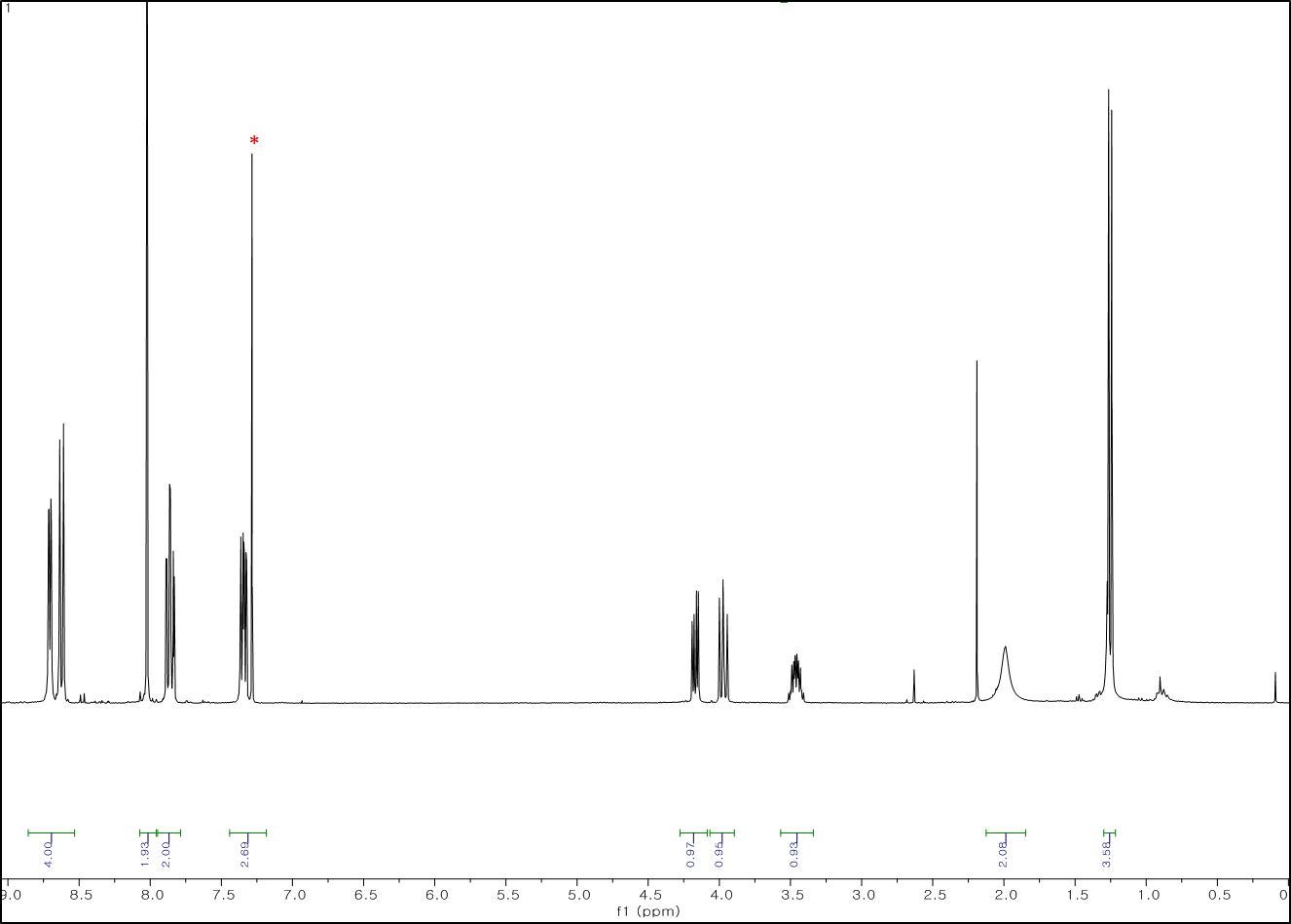


^1^H NMR spectrum (300 MHz) of **3*R*** in DMSO-*d*_6_ at 25 °C.

^13^C NMR spectrum (75 MHz) of **3*R*** in DMSO-*d*_6_ at 25 °C.

^1^H NMR spectrum (300 MHz) of **2*R*** in DMSO-*d*_6_ at 25 °C.

**
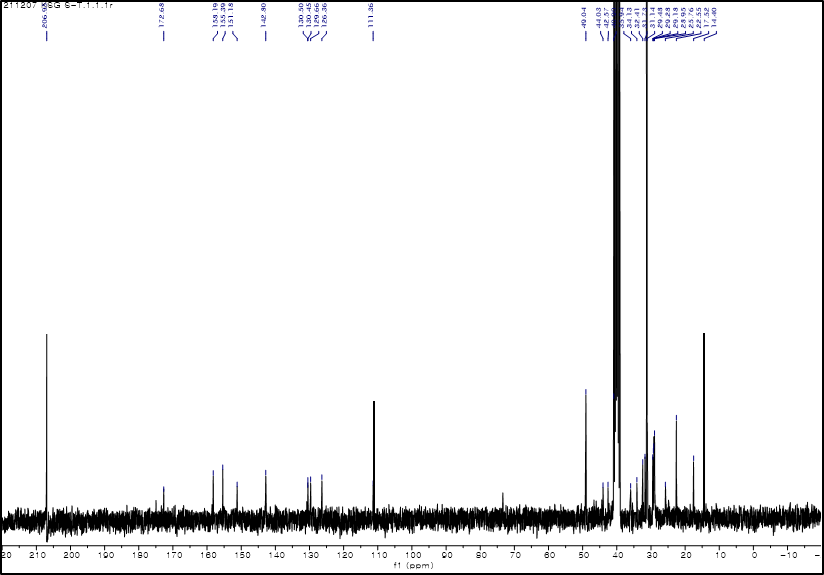
**

^13^C NMR spectrum (75 MHz) of **2*R*** in DMSO-*d*_6_ at 25 °C.

^1^H NMR spectrum (300 MHz) of **L-Pt-Cl** in DMSO-*d*_6_ at 25 °C.

^13^C NMR spectrum (75 MHz) of **L-Pt-Cl** in DMSO-*d*_6_ at 25 °C.


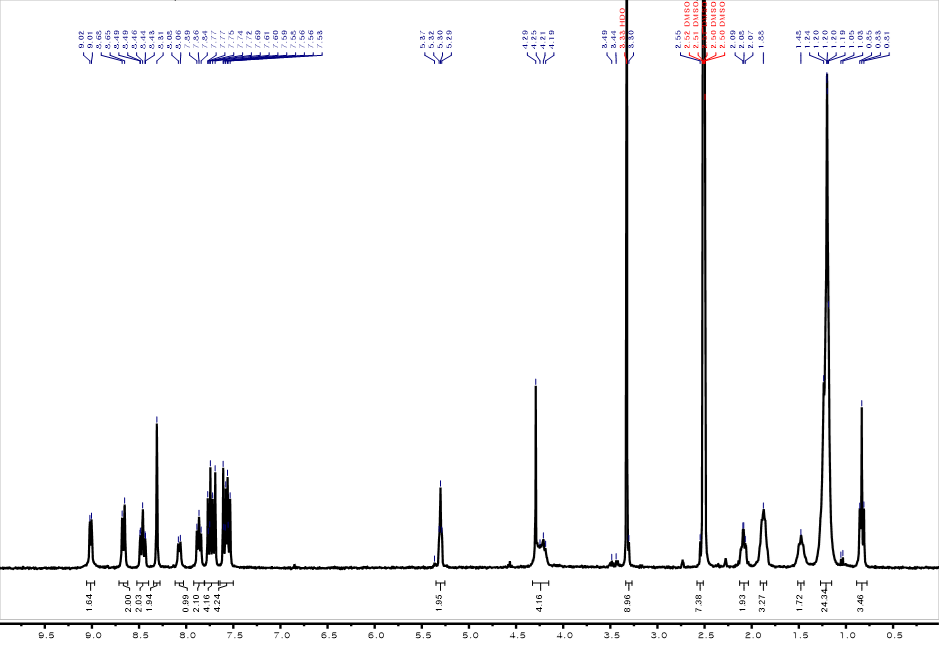


^1^H NMR spectrum (300 MHz) of ***R*-L-Pt-BP** in DMSO-*d*_6_ at 25 °C.

**
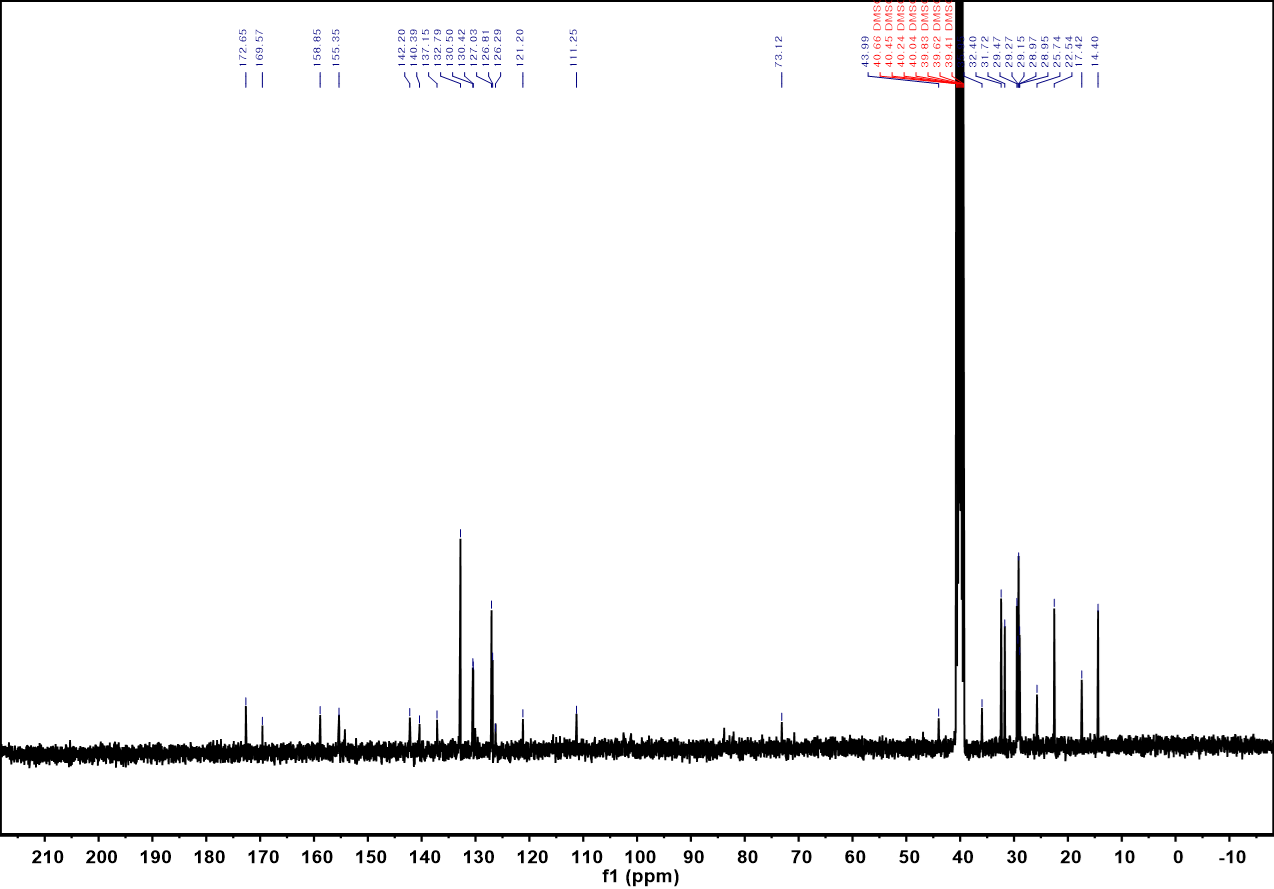
**

^13^C NMR spectrum (75 MHz) of ***R*-L-Pt-BP** in DMSO-*d*_6_ at 25 °C.

**4.2 Mass Spectra**


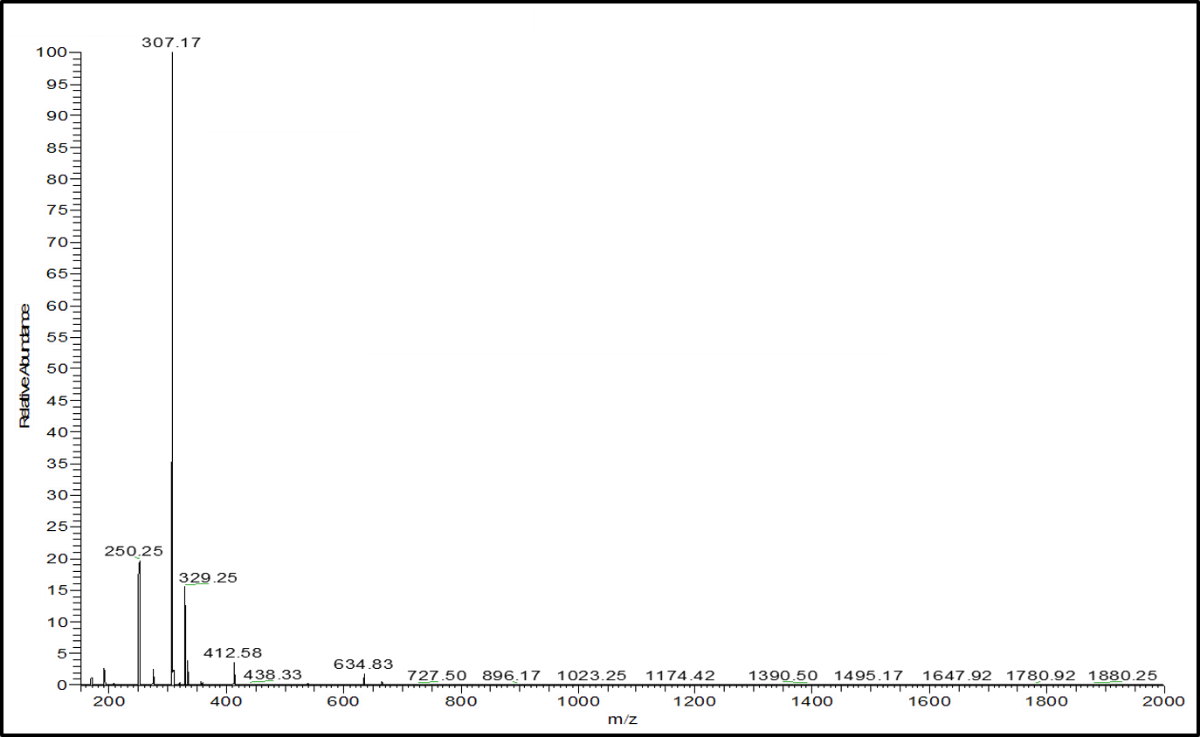


ESI-MS spectrum of **3*R*** in DCM.


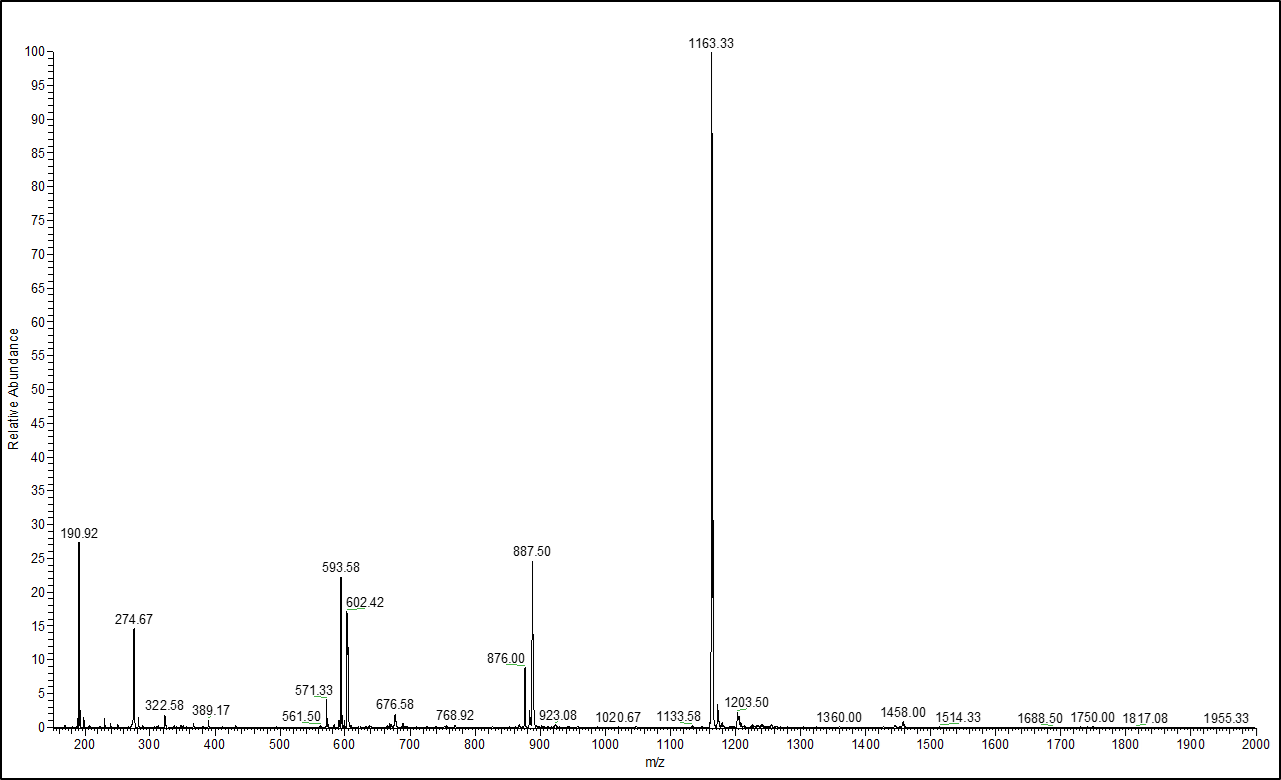


ESI-MS spectrum of **2*R*** in MeOH.


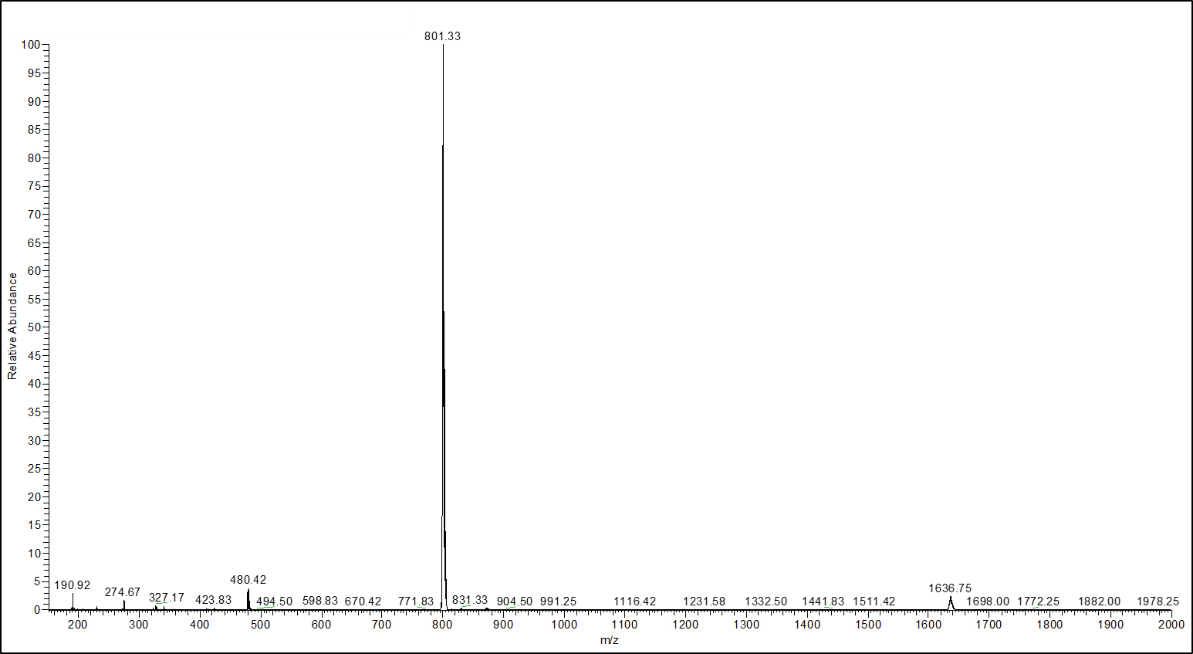


ESI-MS spectrum of **L-Pt-Cl** in MeOH.


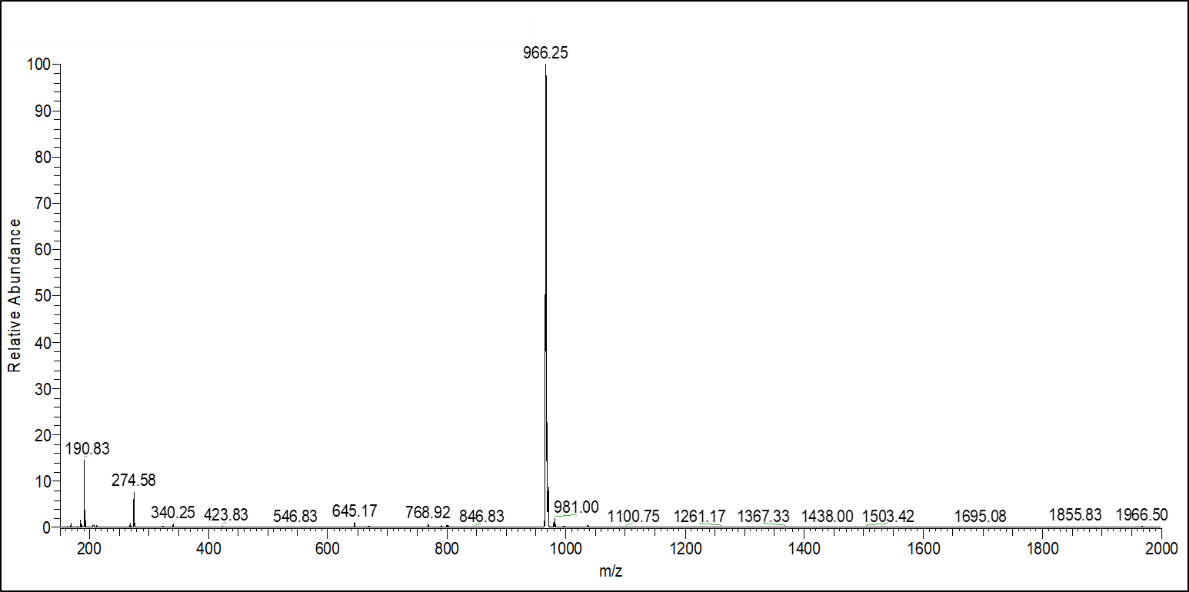


ESI-MS spectrum of *R***-L-**Pt-BP in MeOH.

**5. Supplementary References**

[1] M. M. J. Smulders, M. M. L. Nieuwenhuizen, T. F. A. de Greef, P. van der Schoot, A. P. H. J. Schenning, E. W. Meijer, *Chem.-Eur. J.* **2010**, *16*, 362-367.

[2] P. Jonkheijm, P. van der Schoot, A. P. H. J. Schenning, E. W. Meijer, *Science*. **2006**, *313*, 80-83.

[3] H. Choi, S. Ogi, N. Ando, S. Yamaguchi, *J. Am. Chem. Soc.* **2021**, *143*, 2953-2961

[4] L. Bentea, M. A. Watzky, R. G. Finke, *J. Phys. Chem. C* **2017**, *121*, 5302-5312.

[5] A. M. Morris, R.G. Finke, *Biophys. Chem.* **2009**, *140*, 9-15.
